# Report of Annual Meeting of the American Society of Dental Surgeons

**Published:** 1852-10

**Authors:** 


### *Report of Annual Meeting of the American Society of Dental Surgeons.*

The thirteenth annual meeting of the American Society of Dental Surgeon convened at the Ocean House, Newport, R. I., Tuesday, Aug. 3d, at 10 o'clock, A. M.

The meeting was call to order by the Pres't. Dr. Eleazor Parmley, of New York.

Dr. C. O. Cone, of Baltimore, Secretary of the Society, read the minutes of the last meeting, which were approved.

Dr. E. Townsend, of Philadelphia, offered the following resolution, which was adopted, viz:

*Resolved*, That all gentlemen, in the profession, who may be present, although not members of this society, are invited to attend the meetings of this Association.

A memoranda of the correspondence of the Corresponding and Recording Secretary of the Society, for the last year, was read by Dr. Cone.

Report of the Executive Council, and examining Committee on the order of business was called for, and read.

Dr. Cone tendered his resignation as a member of a committee of the society appointed to collect facts, and report on the subject of irregularities of teeth.

Dr. J. Parmley, offered the following resolution, which was adopted.

*Resolved*, That in consequence of the non-reception of certain papers which the society understand were forwarded by Dr. Dwinelle, bearing upon his case, and which have failed to arrive in time for the action of the society, Dr. Dwinelle's case be laid over until the next annual meeting.

The report of Treasurer read, and referred to auditing committee.

Committee on amendments to the constitution were discharged, and a new committee appointed to draught a new constitution and by-laws for this society.

Report of committee on dental irregularities postponed.

Committee on microscopic examinations, reported progress, and were continued for another year.

Committee on foreign dental literature not present. Adjourned till to-morrow morning.

Aug. 4th.—Meeting called to order by the President. Several members arrived since yesterday. Minutes of yesterday read and approved.

Committee on dental irregularities called for. Dr. Bridges, of Brooklyn, chairman, reported, that in consequence of ill health, had not been able to attend to the duties of said committee. Dr. J. Allen, had no report in form—expected to have had an opportunity of conferring with other members, and thus concentrating a report. But as no such opportunity had been afforded him, was unable to present a report. Whereupon, a short discussion arose, and on motion, the committee were dissolved for the purpose of appointing a new one.

Dr. J. D. White, of Philadelphia, being called upon, read to the Society, a paper on the Anatomy and Physiology of *Dentine.* This was a tersely written paper, evincing minute and critical attention to the subject which he had taken in hand. We hope soon to have the pleasure of laying it before our readers.

Committee on Townsend's resolutions, reported a series of resolutions touching the necessity and propriety of Dental collegiate instruction, and the establishment of lectures, and professors of this speciality, in the medical schools of this country, which gave rise to quite a spirited discussion, after which the resolutions were laid on the table.

The committee on practical dentistry, asked to be discharged from their further duties, as such committee, and stated as a reason, that the dental journals and periodicals of this country, were superceding, by their able and judicious publications, any report from this committee, on the subject of Practical Dentistry. They were accordingly discharged.

Dr. E. Townsend, read a paper on the subject of *Professional Fees.* This paper was written in the characteristically elegant style of Dr. T. and the subject is worthy the attention of the dental profession. We are promised a copy for publication in the Recorder.

Dr. E. J. Tucker, of Boston, read a paper on the subject of *dental irregularities*, and fruitful of instruction upon the subject treated, containing many hints of importance to the profession. This paper also, we hope to publish in the Recorder.

Dr. Robertson, of Manchester, N. H., read an essay upon the subject of the influence of diseased teeth and gums upon the general health of the system, a copy of which he has kindly furnished us for publication, and which, when published, we would be glad to see in the hands of every practitioner of medicine.

The thanks of the society were voted to Drs. White, Townsend, Tucker, and Robertson, for their several papers, and copies requested for publication.

On motion of Dr. Hullihen, of Wheeling, Va., voted a gold medal, of the value of $25,00 be awarded by this society for the best paper upon the cause and treatment of irregularities of the teeth, to be read at the next annual meeting of this society.

The election of officers, resulted as follows,

*For President.*—Eleazor Parmley, New York.

1*st Vice President.*—E. Townsend, Philadelphia.

2*d Vice President.*—J. H. Foster, New York.

3*d Vice President.*—J. Tucker, Boston.

*Cor. and Rec. Secretary.*—C. O. Cone, Baltimore.

*Treasurer.*—E. J. Dunning, New York.

*Librarian.*—D. R. Parmley, New York.

Publishing, Executive, and Examining committees, re-elected.

Drs. Cone, Lord, Hullihen, Townsend, Dunning, and Bridges, were appointed to prepare essays, to be read at the next annual meeting of the society.

The society voted thanks to Messrs. Jones, White, and McCurdy, for their enterprise and liberality, in so promptly and handsomely reporting the doings of the previous meeting at Philadelphia.

---

### AFTERNOON SESSION.

Dr. C. O. Cone, of Baltimore, read a paper before the society upon the subject of a new mode of treating exposed dental nerve.

The subject was first brought to the attention of Dr. Cone, by a communication from Dr. Hullihen, of Wheeling, Va., who first discovered, and practised the operation some three years ago. At the instance of Dr. H., Dr. Cone, had been experimenting for sometime past, and minutely detailed his operations and their results to the society in the paper above referred to. And also laid before the society a paper from Dr. Hullihen, relating to the same subject. This subject elicited many questions, and considerable discussion, and the operarion by many is regarded as one of the most important steps to which the profession has advanced, in the practice of dental surgery; and the commencement of a new era in its history. The operation consists, in drilling into the nerve cavity about a line above the margin of alveoli, through the gum, and alveoli—*without separating the nerve*, and wounding it as lightly as possible. The drill should be *spear-shaped*—with one cutting edge longer than the other—shaft smaller than the drill head, and driven with a bow, with slack string, and the size of the drill to be the same as the size of the nerve, at the point where the fang is perforated.

The operation is to be performed where the nerve is exposed, in excavating the crown, so as to produce pain and pressure, or, in that class of operations, where the nerve is usually destroyed with arsenic. The results of this operation are said to be the preservation of the vitality of the nerve, instead of its destruction, as in cases where arsenic is used. While at the same time, the operator is enabled to fill the cavity in the crown, without pain, or inconvenience.

Much care is requisite for the success of this operation, and the drill cuttings must be carefully removed from the fang, where the hole is drilled.

This opening, it is said, will fill up with new ossific matter, and the tooth become as sound and healthy as if it never had been wounded at all.

Such, in brief, is our very imperfect description of an operation which opens a new field for experiment, with a fair prospect of success. We expect by no means to do justice to the very able and elaborate report upon this subject, and presume to give the readers of the Recorder merely a general idea of the operation. We hope soon to be able to lay the matter before them in a more complete manner, by publishing the papers above referred to, meantime they must be content with our scraps of memory.

Dr. J. D. White asked the question, whether the object of puncturing a tooth, was to allow the escape of extravasated blood, while at the same time, it admitted a sufficient amount of nerve in the canal, to maintain the vitality of the crown? Dr. Hullihen was not prepared to decide the question, but made some suggestions as to the probable physiology of the operation.

After considerable miscellaneous intercourse between members, the society adjourned to meet at Westpoint the second Tuesday in August next, at 10 o'clock A. M.—*Dental Recorder.*

